# The Medical Council

**Published:** 1898-06-04

**Authors:** 


### THE MEDICAL COUNCIL.

The sixty-fifth session of the General Medical Council commenced on Tuesday, the 24th inst, under the President (Sir William Turner); and the first business was to receive an intimation from the Privy Council that Mr. Charles Sissmore Tomes, F.R.S., F.R.C.S., L.D.S., had been appointed for five years as a Crown representative, in the vacancy occasioned by the death of Sir Richard Quain. Mr. Tomes was introduced by Mr. Bryant, and received by the President in the accustomed manner. The President then delivered an introductory address, dealing with the business to be transacted during the session, for which address he received the thanks of the Council, and it was ordered to be entered on the minutes. A letter was read from the family of the late President, acknowledging the vote of condolence of the preceding session; and a letter was also read from Miss Quain, offering to the Council, on behalf of her sisters and herself, the marble bust of the late Sir Richard Quain, by Mr. Brock, R.A., which was exhibited last year in the Royal Academy. The gift was gratefully accepted, and a suitable resolution of thanks was passed on a subsequent day, after the bust had been received. An expression of participation in the national regret at the decease of Mr. Gladstone was moved from the chair and carried unanimously; a Business Committee for the session was appointed; and the customary tables showing the results of examinations were presented and acknowledged.

Mr. Victor Horsley was then afforded an opportunity of calling the attention of the Council to what he described as an "infringement of the rights and privileges of the members of the Council by the recent action of the President in directing the Registrar to refuse a member the exercise of his right of inspection of documents, the property of the Council." The President had already referred to this matter in his address; and, after Mr. Horsley had spoken, an opinion by Mr. Muir Mackenzie was read. Mr. Mackenzie stated in substance that the right claimed by Mr. Horsley did not exist, and that no right of any kind had been infringed. Documents which were the property of the Council were not the property of individual members; and access to them could only be obtained by permission of the Council itself, or of its Executive Committee, given by formal resolution in reply to an application. It was resolved that "the Council, having heard the President's statement as to the legal ground of his action in reference to Mr. Horsley's application, expresses its complete satisfaction therewith, and passes to the next business on the programme." On a subsequent day the matter was finally dealt with, and Standing Orders were passed to the effect that a member of Council desiring access to documents must apply to the Council itself, to the Executive Committee, or to the President in the intervals between sessions, stating the nature of the documents required, as well as the objects of the applicant; and further providing that no information obtained from such documents should be made public until it had first been laid before the Council. Mr. Horsley then moved a resolution for the appointment of a committee to "inquire into the conduct of the legal business of the Council in the past and at present, and the cost to the Council incurred thereby." A debate ensued, which led to expressions of warm admiration on the part of the more experienced members of the manner in which the legal business had always been conducted; and the resolution was defeated by a two to one majority. It was then five o'clock, and the Council adjourned for the meetings of committees.

On Wednesday Mr. Horsley again opened the ball by a motion that "the late president's letter of July 1st, 1897, addressed to the Privy Council on the matter of medical practice in Italy, and the publication advertised by the Council, entitled 'Reciprocity of Medical Practice in Relation to Foreign Countries,' be and are hereby rescinded and withdrawn." Mr. Horsley was permitted to withdraw his motion in favour of a modification of it, which was lost; but the discussion showed that the position of affairs had undergone some changes since the memorandum on reciprocity was issued in 1893, and it was therefore resolved that this memorandum should not be reprinted without further orders. The resignation of Mr. Chance, one of the Assistant Examiners in Surgery to the Apothecaries Hall of Ireland, was received, and a committee was appointed to consider the selection of a successor, a proceeding which, on a subsequent day, led to the appointment of Mr. Howse, Surgeon to Guy's Hospital. A committee was also appointed to meet delegates from the Midwives Bill Committee, and to hear their proposals for future legislation on the question, the latter committee having expressed a wish to make such alterations in the present Bill as would secure for it the support of the Council.

On Thursday, the whole day was occupied in the consideration of penal cases. Mr. Masson and Mr. Mostertz, both of whom had been found in the last session to have committed the offence of "covering," although not in a very aggravated form, and whose cases had therefore been adjourned, both attended and satisfied the Council that they had abandoned the practices complained of, on which showing it was resolved that no further steps should be taken against them. Mr. George Hamilton Wyse, who was left last session in a similar position, was the subject of renewed complaint from the Wigan Medical Guild, and his name was ordered to be erased from the Register. The same fate befel Mr. Joshua Hamilton Hart, whose offences are of sufficient interest to be made the subject of observations in another column.

A very important series of memorials and petitions, from various bodies and individuals, having reference to the recent decision of the Council no longer to permit the employment of unqualified assistants for the discharge of medical responsibilities, had been received during the recess; and all of them were referred to a committee, with Mr. Teale as chairman, and composed of Drs. Church, MacAlister, and Bruce, and Messrs. Bryant and Browne, with instructions to report during the current session.

On Friday, a letter was received from the office of the Earl Marshal, expressing regret that the limits of space in Westminster Abbey rendered it impossible for him to accede to a request for admission of members of the Council on the occasion of the funeral of Mr. Gladstone; and it was thereupon resolved that the Executive Committee should take the steps necessary to secure that the Council shall receive due recognition and representation in national functions and ceremonies. A report was received from the Committee on Medical Aid Associations, stating that conferences from which good was expected had been held with various persons, and recommending a period of patience and of endeavours at mutual conciliation to all who were concerned. The Council went into committee for the consideration of a report on dental education and examination, and had not concluded when the time for adjournment arrived.

On Saturday the whole of the business was transacted *in camerâ*, and only the results can be reported, with the single exception that Mr. Brown obtained the permission of the Council, in deference to legal advice, and in consequence of the questions at issue being still *sub judice*, to withdraw certain notices of motion which he had placed upon the programme with reference to the case of Mr. H. K. Hunter, who had been prosecuted by the Council for the improper use of certain titles, and at whose request a case for the consideration of the Queen's Bench Division had been stated by the magistrate who convicted him. The Council ordered certain prosecutions, placed upon the minutes a letter from Mr. Upton (the solicitor to the Society of Apothecaries), on the subject of the power of the Society to restore the name of a person who had been removed from their list of licentiates, and refused registration in the Dentist's Register to four applicants—Messrs. Merrill, Bush, Oldfield, and Royce—whose cases have been previously considered and are now finally disposed of. Mr. Allen was re-elected registrar, and the Council adjourned.

On Monday the Council passed a resolution suggested by the Penal Cases Committee, to the effect that no proceedings should be taken, in any complaint as to the conduct of a registered practitioner, until the complainant had put his allegations into the form of a statutory declaration. It then went into committee to consider a series of recommendations with regard to the education and examination of dental students, and was occupied with them for the remainder of the sitting.

On Tuesday the business was of a very miscellaneous description, and the Council sat until nearly eight o'clock in order to bring the session to a close. Something like an hour was occupied, even if not wasted, in an exchange of explanations between Mr. Bryant, the chairman of the Dental Education and Examination Committee, and two Scottish representatives who were aggrieved at his account of the action of their respective bodies. Mr. Brown had given notice of four resolutions of a somewhat revolutionary character, but thought it more prudent to withdraw them all, as he was not able to find a seconder for the three last. Mr. Horsley proposed a resolution removing the control of dental matters from the Executive Committee, and this was carried by nine votes against eight, thirteen members abstaining from voting. Mr. Horsley had seven more resolutions on the paper; but, on the motion of Dr. Atthill, seconded by Mr. Teale, the whole of them were referred to the Executive Committee for consideration and report, so that they may be dealt with collectively in a future session. Reports were received from the Education Committee, the Committee on Fines under the Medical Acts, the Finance Committee, the Pharmacopœia Committee, and the Student's Registration Committee, all dealing with almost routine business. The Pharmacopœia Committee recorded the completion of the work on which it has been engaged for the last five years, and the sale, in the first fortnight, of over eleven thousand copies of the book; and resolutions of thanks, to the committee itself, and to the various scientific men and bodies from whom it had received assistance, were moved from the chair. There was also a report from a committee which had been appointed to consider a number of applications to the Council for some temporary relaxation of its recent determination to put a stop to the employment of unqualified assistants for the discharge of distinctly medical duties; and this report, which concluded with the following recommendations, was unanimously adopted by the Council. The questions raised by the memorialists were found to fall under four heads, namely:

"(1) Whether the Council can, consistently with the law, authorise the examining bodies to hold special examinations for a particular class of persons who have lately been employed as unqualified assistants?

"(2) Whether the Council is prepared to suspend the operation of the resolution adopted on November 24th, 1897, in the interest of certain practitioners, who state that they are at present unable to obtain qualified assistance in their practice?

"(3) Whether the Council can take any steps for the reinstatement of certain unqualified assistants, who have been dismissed by their employers since the issue of the Council's notice?

"(4) Whether the Council should issue any detailed definitions or explanations as to the extent to which unqualified persons may be 'legitimately employed' by medical practitioners?"

The answers given to these questions by the committee, and adopted by the Council, are:—

"(1) The Council has no power under the Medical Acts to request or authorise any of the licensing bodies to institute special examinations for a registrable license, other than the 'qualifying examinations' in medicine, surgery, and midwifery, defined by the Act of 1886.

"(2) There appears to be evidence that at the present moment certain practitioners do find difficulty in obtaining the services of legally qualified assistants. Junior practitioners are no doubt unwilling in many cases to accept the inferior and ill-paid positions which have hitherto been occupied by unqualified men. But it is reasonable to expect that before long improvement in the terms and conditions offered by principals, and the natural operation of the economic law of supply and demand, will remove the difficulty complained of. The Council cannot regard such a temporary inconvenience as a ground for suspending the operation of a resolution deliberately adopted by it, with a view to the repression of fraudulent and dangerous practices.

"(3) It is not within the province of the Council to take any steps in the direction suggested.

"(4) The terms of the notice issued by the Council convey with sufficient clearness the limitations which it was intended to place upon the employment in connection with medical practice of unqualified persons by registered practitioners.

"The registered practitioner will be liable to proceedings before the Council:—

"(a) If he knowingly allows 'such unqualified persons to attend or treat patients in respect of matters requiring professional discretion or skill.'

"(b) If he thus allows 'such a substitution of the services of an unqualified person' for his own, this substitution being 'in its nature fraudulent and dangerous to the public health.'

"(c) If, under cover of his own qualifications, he knowingly does anything to enable 'an unqualified or unregistered person to attend or treat any patient, to procure or issue any medical certificate or certificate of death, or otherwise to engage in medical practice, as if the said person were duly qualified and registered.'

"The employment of unqualified 'dressers, midwives, dispensers, and surgery attendants' can be 'legitimate' only if it does not come under one of the foregoing heads, and when it is carried out 'under the immediate personal supervision of registered medical practitioners.'

"The Council do not think it necessary or expedient to issue more precise definitions, or to lay down regulations intended to meet all the particular cases which may arise. As in all other instances, the Council must deal with each case on its merits.

"Whenever a practitioner is alleged to be making improper use of unqualified assistance, the Council will have to be satisfied that the allegation is fully established."

The adoption of these important recommendations practically concluded the business of the session. Mr. Horsley moved two resolutions expressive of his views, but both were rejected. The various committees for the ensuing year were then elected, and the Council rose.

